# Impact of scientific production of Italian scientists in exercises and sport sciences by measuring the author-weighted *h*-index

**DOI:** 10.3389/frma.2024.1466811

**Published:** 2024-11-01

**Authors:** Gaetano Raiola, Giovanni Esposito, Rosario Ceruso, Francesca D'Elia, Tiziana D'Isanto

**Affiliations:** ^1^Research Center of Physical Education and Exercise, Pegaso University, Naples, Italy; ^2^Department of Human, Philosophical and Education Sciences, University of Salerno, Fisciano, Italy; ^3^Department of Neuroscience, Biomedicine and Movement, University of Verona, Verona, Italy

**Keywords:** scientific production, sport sciences, scientific disciplinary sectors, Scopus, citations

## Abstract

This study aimed to measure the consistency of the impact of scientific production, weighted by authorship, of Italian scientists in two academic disciplines (AD) of Exercise and Sports Sciences (ESS) from 2017 to 2022, with a specific focus on the subfield “Sport Sciences”, using topic-specific keywords. Through the Scopus database, the scientific products of Italian ESS scientists associated with each keyword were identified. Subsequently, total and relative metric parameters from 2017 to 2022 were collected, including the total and relative number of citations. To evaluate the impact of the publications, the total and relative *h*-index were calculated, and weighted by considering different categories of authorship. Specific weights were attributed to each category: single author, first author, last author and co-author, following the classifications already in use on Scopus for each author. The trends of total and relative metrics, including citations and *h*-index, from 2017 to 2022 were analyzed using Spearman's correlation. Non-parametric linear regression analysis was used for the predictive analysis of these trends. Among the 83 identified ESS scientists, a detailed analysis revealed that 31.3% were full professors, 42.1% associate professors, and 26.6% researchers. Less than half of these scientists were directly affiliated with ESS. Despite minority representation, significant positive correlations emerge between total and relative citations from 2017 to 2022 (*r* = 0.687) and between the weighted total *h*-index and the weighted relative *h*-index (*r* = 0.965). Significant trends emerge in the metric parameters of the same scientists when analyzed separately by AD. The regression results indicate that variations in total citations and the weighted total and relative *h*-index can predict or explain the observed changes in 2017–2022 *(p* < 0.05). This result suggests that the production and impact of research in the field of ESS follow the same general trend as production and impact in the specific subfield.

## 1 Introduction

In Italy, research on the scientific production of Exercise and Sports Sciences (ESS) scientists has only recently begun. Previous studies have examined the correspondence between the titles of the most highly cited scientific products in the Google Scholar database of Italian scientists in the two academic disciplines (AD) of “Physical Training and Methodology” (code M-EDF/01) and “Sport Sciences and Methodology” (code M-EDF/02) and their respective declarations (D'Isanto et al., 2023, 2024). Both AD are part of the broader ESS scientific disciplinary group, each with a specific focus. While M-EDF/01 primarily addresses educational, wellness, and health goals related to physical activity, M-EDF/02 is more oriented toward sports science, particularly in the assessment of sports performance and athletic optimization. The analysis showed that the scientific production of academics framed in the AD of M-EDF/02 had greater affinity with topics of the biomedical area, while those in M-EDF/01 had greater affinity with the pedagogical area. This trend could be attributed to the significant role that “related” scientific knowledge plays in shaping the scientific identity of scientists within the two AD of M-EDF. However, these affinities do not always reflect consistency with M-EDF's topics.

Twenty-six years after the recognition of ESS at the university rank (Italy, 1999), it is now useful to measure the impact, i.e., the influence and relevance of the scientific production of Italian ESS scientists on a global scale (Aragón, 2013; Dardas et al., 2023). This is possible using the basic model of classification of scientific knowledge, currently adopted by the international scientific community characterized by domains, fields and subfields adopted by Science-Metrix (Rivest et al., 2021). This ranking model has generated the World's Top 2% Scientist list, which is the global ranking of scientists with the highest level of scientific productivity developed by Stanford University (Ioannidis et al., 2019), in collaboration with Elsevier and the Scopus database (Ioannidis et al., 2020, 2021; Ioannidis, 2022, 2023). For each scientist, the field in which they were active, along with the corresponding ranking, citations and *h*-index, were indicated. The composite index obtained made it possible to measure the impact in citation terms of each scientist and each paper by applying corrective factors, related, for example, to self-citations, single-authored papers and their corresponding ranking, as well as the metric dynamics of each scientific area. However, the database does not provide country-disaggregated citation data on scientists active in the ESS. Consequently, it is not possible to assess the overall impact of specific research conducted by Italian scientists in this disciplinary subfield.

Italy has recently adapted its knowledge classification model, characterized by the 14 scientific areas defined by the National University Council (CUN), to the international one. With the proposal of 28 September 2016, it developed a list of keywords related to scientific-disciplinary indicators useful for identifying the profile of scientists (CUN, 2016). Scopus database feature analysis revealed that such keywords were probably used to classify the individual subfields on which Ioannidis et al. (2019) conducted their studies of the top 100.000 scientists. Through the “Researcher Discovery” search function of Scopus Elsevier, it is possible to extract data on scientific production and its impact on the international scientific community as it applies to the Italian peculiarity of classifying scientific knowledge through the scientific disciplinary declarations of AD. This approach aims to provide a more accurate and comprehensive view of the citation impact of Italian scientific production through a classification of authors based on the results pertaining to their scientific field, initially conducted only for specific scientific production (D'Elia et al., 2023). A previous study (Raiola et al., 2024), analyzed the impact of the scientific production of Italian ESS scientists, focusing on the subfield “Sport Sciences” using some keywords characterizing the subfield. From that study, data, extracted from the Scopus database pertaining to the period 2017–2022, included information on articles, citations, and *h*-index of the top 200 Italian scientists. It was found that the overall production and impact of the scientists followed the trend of those related to the subfield, indicating proportionality in overall research as opposed to research focused on specific keywords.

Currently, in Italy, there is still a lack of systematic studies that assess the citation impact of ESS scientists according to international standards weighted by authorship. The number of scientific products and the impact on the international community for the subfield “Sport sciences” should be weighted according to the contribution made by individual authors, to eliminate the generality of intellectual property attributed to all signatories of a scientific article. The generic classification of authors' scientific weight based only on the *h*-index relative to the subfield is not accurate, because it does not consider the different contributions of single, first, last or co-author (Batista et al., 2006). Moreover, the possibility offered by Scopus to distinguish position in authorship is not sufficient to solve this problem. Therefore, the aim was to measure the consistency of the impact of scientific research by Italian scientists within the ESS framework during the reference period 2017–2022. This assessment specifically pertains to their analytical contributions in collaborative works within the subfield of “Sport Sciences”, encompassing keywords such as “Sports,” “Physical education,” “Physical training,” “Physical exercise,” “Sport education,” and “Sports science,” in comparison to the overall impact of scientific production.

## 2 Method

The analysis of the scientific production of Italian scientists, differentiated by roles and functions (Full Professors, Associate Professors, and Researchers), within the academic disciplines (AD) of Physical Training and Methodology (code M-EDF/01) and Sport Sciences and Methodology (code M-EDF/02), concerning the six keywords previously identified within the subfield, required several stages of processing. The selection process for these six keywords was guided by specific criteria. First, a qualitative analysis was conducted to evaluate the relevance and uniqueness of each keyword, considering its applicability in existing research and publications. Aspects such as adherence to prevailing research topics and influence within the field of knowledge were considered. Additionally, co-occurrence frequencies of the keywords were examined, identifying those with the least conceptual overlap with other keywords in the database. This ensured that each selected keyword represented a distinct area of study, avoiding redundancies. During this process, the Researcher Discovery feature of Scopus was utilized, allowing for keyword searches within the database and directly linking to relevant researchers and documents. Additionally, the same feature was employed to analyze the six keywords characterizing the subfield. Specifically configured searches were employed to retrieve only Italian authors who have produced works associated with each keyword, ensuring their inclusion in the analysis. Data collection was conducted at the end of 2023. In accordance with the features of Scopus Researcher Discovery, the analysis focused on the period from 2017 to 2022. This timeframe was selected for two primary reasons: first, to assess the most recent developments and trends in scientific production within these academic disciplines, thereby reflecting the evolving nature of the field; and second, to comply with the limitations associated with the functionality of Scopus Researcher Discovery, which facilitates targeted analysis of relevant and up-to-date data. After setting the country-of-interest filter, the software automatically provided up to 200 Italian authors associated with each searched keyword. Among these authors, ESS scientists framed within one of the two AD were identified. In Italy, the total number of such scientists amounts to 259 (141 in M-EDF/01 and 118 in M-EDF/02).

In the second stage of the analysis, after identifying Italian ESS scientists, the following metrics were collected for the period 2017–2022:

- *Total citations* = include all citations obtained by an author, regardless of the specific field, including studies unrelated to the subfield “Sport Sciences”. The total citation index was calculated by summing all citations received across the entire body of work produced by each author during this period.
- *Relative citations* = refer only to publications related to one of the six keywords, thus determining the six “relative” indexes associated with each keyword. The relative citation index was calculated by summing the citations obtained from the products associated with each of the six selected keywords during the same timeframe.

Subsequently, the weighted total *h*-index and weighted relative *h*-index related to each keyword were calculated for each of the scientists framed in the ESS. The process to calculate the authorship-weighted *h*-index involves finding the *h*-index for different categories of authorship: single author (*h*-single), first author (*h*-first), last author (*h*-last), co-author (*h*-coauthor). This is done by sorting the publications within each category by the number of citations and determining the point at which the number of citations reaches or exceeds the number of the publication itself. The *h*-indexes obtained for each category were used in the weighted *h*-index formula, where specific percentage weights were assigned to each category, reflecting the relative contribution of each authorial position to the overall scientific output. These weights represented each author's contribution based on their position in each article, as defined by Scopus Elsevier. They were derived from an analysis of literature and common practices in the field of scientific research, where an author's position often indicated their level of involvement and responsibility in the research project. The weights were assigned according to these specific criteria:

- *Single author*: 100%, since he is responsible for all aspects of the study, from design to implementation
- *First author*: 50%, as he contributes substantially to the initial design and conduct phase of the study
- *Last author*: 40%, since he usually provides an overview and guides the entire research work
- *Co-author*: 5%, as the contribution can vary considerably from second to penultimate author.

An author's total authorship-weighted *h*-index was calculated by summing the products of the *h*-indexes for each authorship category, multiplied by their respective percentage weights, for all the author's publications during 2017–2022. Similarly, the values of the *h*-indices for each authorship category related to the six keywords were summed for each scientist. Next, the *h*-index quotient related to the six keywords, multiplied by their respective percentage weights, was calculated for all the author's publications. Calculating the numerical quotient for the *h*-index offers several methodological advantages. First, it makes it possible to normalize relative *h*-index values concerning the total number of keywords considered, thus reducing the risk of bias in results due to differences in the number of publications and citations across search fields. This approach allows for a weighted *h*-index that effectively represents the author's scientific impact over time. In addition, the ease of interpretation of the numerical quotient makes it an accessible tool for evaluating the author's performance clearly and intuitively. This facilitates comparisons between authors and offers a more coherent view of scientific impact over time.

### 2.1 Statistical analysis

Descriptive statistics (mean ± SD) were utilized to summarize the data for the different variables. The Shapiro-Wilk test indicated that the data did not follow a normal distribution, providing an initial insight into the nature of the data. Trends between total and relative metrics weighted by authorship (citations and *h*-index) during the period 2017–2022 were assessed using Spearman correlation. Analyzing the correlations between weighted total *h*-index and relative citations, as well as between weighted relative *h*-index and total citations, reveals the dynamic relationship between these variables. Consequently, if a trend emerges indicating a significant relationship, it implies that the overall quality of research output aligns with relative citations, highlighting a close connection between the researcher's specific expertise and their extensive production. Likewise, the correlation between weighted relative *h*-index and total citations underscores this procedural consistency. Finally, predictive modeling of trends was performed through non-parametric linear regression analysis. The significance level was fixed at *p* ≤ 0.05 using Statistical Package for Social Science software (Version 28.0, IBM SPSS Statistics, Chicago, IL, USA).

## 3 Results

Among the 200 Italian scientists returned by the Scopus Researcher Discovery function, a total of 83 researchers framed on ESS emerged with the following distribution: 26 Full Professors = 31.3%, 35 Associate Professors = 42.1%, and 22 Researchers = 26.6%. The disaggregated data for AD is as follows: 17 Full Professors, 17 Associate Professors, and eight Researchers for the AD “M-EDF/01”, and nine Full Professors, 18 Associate Professors, and 14 Researchers for the AD “M-EDF/02”. The tables below present the analysis of the impact of the total and relative scientific production of Italian ESS scientists, distinctly categorized by roles and functions in aggregated form (Table 1) and distinctly for AD in disaggregated form (Table 2).

**Table 1:** Impact of the scientific production of ESS scientists aggregated for AD.

**Authors information**	**Total citations 2017–2022**	**Total weighted *h*-index 2017–2022**	**Relative citations 2017–2022**	**Relative weighted *h*-index 2017–2022**
Full professors (*n* = 26)	719.9 ± 594.6	14.0 ± 9.5	694.4 ± 596.5	13.5 ± 5.0
Associate professors (*n* = 35)	664.2 ± 788.3	12.3 ± 6.8	544.4 ± 714.9	12.5 ± 11.5
Research scientists (*n* = 22)	433.2 ± 275.8	10.5 ± 5.5	344.7 ± 378.7	10.8 ± 7.0

ESS, exercise and sports sciences; AD, academic disciplines.

**Table 2:** Impact of the scientific production of ESS scientists disaggregated for AD.

**Authors information**	**Total citations 2017–2022**	**Total weighted *h*-index 2017–2022**	**Relative citations 2017–2022**	**Relative weighted *h*-index 2017–2022**
**AD: physical training and methodology (code M-EDF/01)**				
Full professors (*n* = 17)	723.4 ± 685.6	14.8 ± 11.8	745.8 ± 951.6	15.2 ± 15.3
Associate professors (*n* = 17)	518.9 ± 439.3	11.1 ± 7.0	589.1 ± 880.7	11.4 ± 7.5
Research scientists (*n* = 8)	382.7 ± 245.2	8.4 ± 3.7	201.5 ± 187.5	8.1 ± 2.5
**AD: sport sciences and methodology (code M-EDF/02)**				
Full professors (*n* = 9)	735.6 ± 453.9	12.8 ± 7.0	634.1 ± 605.9	10.9 ± 5.5
Associate professors (*n* = 18)	793.7 ± 992.2	13.5 ± 6.4	515.3 ± 501.6	13.5 ± 9.3
Research scientists (*n* = 14)	510.2 ± 328.2	11.7 ± 6.0	446.5 ± 431.5	12.4 ± 8.2

**Figure 1:**
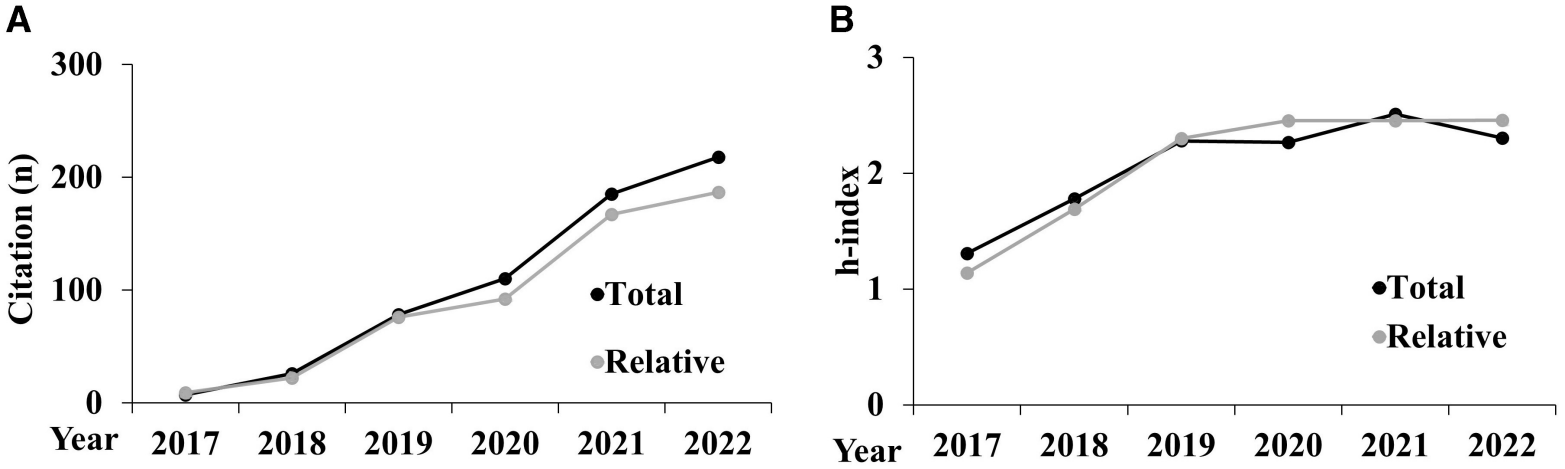
Trends of the citations **(A)** and *h*-index **(B)** analyzed aggregated for AD.

**Figure 2:**
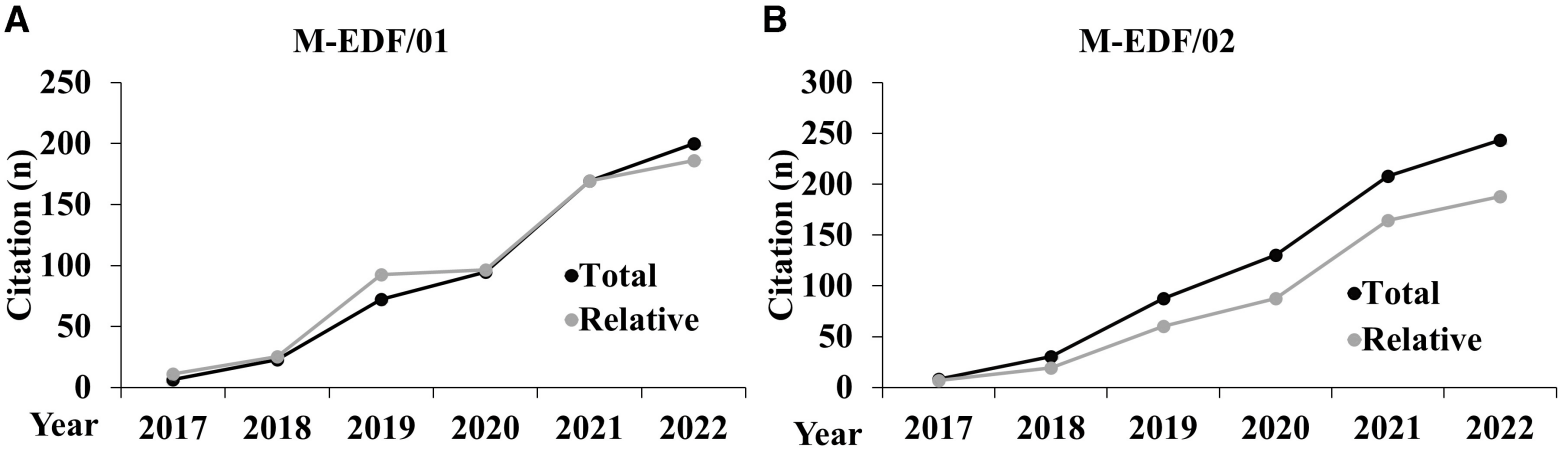
Trends of the citation metrics disaggregated for the AD of M-EDF/01 **(A)** and M-EDF/02 **(B)**.

**Figure 3:**
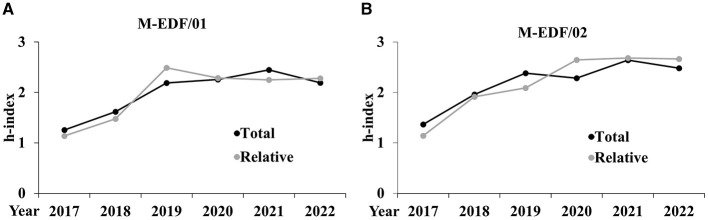
Trends of the *h*-index disaggregated for the AD of M-EDF/01 **(A)** and M-EDF/02 **(B)**.

The following graphs represent the trends of the citations and *h*-index metrics analyzed during the period 2017–2022, both in aggregated form (Figure 1) and disaggregated by AD (Figures 2, 3).

In Figure 1A, while total citations show a sharp increase, relative citations rise more modestly, indicating that growth in the sport sciences is less pronounced. In Figure 1B, the total *h*-index increases steadily, but the relative *h*-index rises more slowly. This divergence suggests that, despite overall improvements, the relative impact of sport sciences research is growing at a different rate compared to broader academic trends, highlighting variations in recognition and influence.

In both subfields, relative citations generally align with total citations but show some differences. For M-EDF/01, relative citations closely track total citations, even surpassing them briefly in 2021, which suggests that the subfield is keeping pace with or slightly outperforming broader citation trends. In contrast, for M-EDF/02, total citations consistently exceed relative citations from 2020 onward, indicating that while citations are increasing in this subfield, they are not growing as fast as in the broader field.

The relative metrics in both ADs show a trend where the specialized research in sport sciences is either matching or slightly outperforming the total metrics in the broader context. Notably, the relative metrics tend to be either aligned or slightly elevated compared to the total, indicating that the subfield's contribution is competitive or growing in visibility and impact within the broader academic or scientific landscape.

To assess the existence of a trend during 2017–2022 among the metrics considered, a correlation analysis was conducted, both in aggregate (Table 3) and disaggregated by AD (Table 4), as shown below.

**Table 3:** Correlations between metrics of ESS scientists aggregated by AD.

		**Total citations 2017–2022**	**Total weighted h-index 2017–2022**	**Relative citations 2017–2022**	**Relative weighted *h*-index 2017–2022**
Total citations 2017–2022	*r*	1	−0.027	0.687^**^	−0.027
	*P*	–	0.812	< 0.001	0.812
	*N*	83	83	83	83
Total weighted *h*-index 2017–2022	*R*	−0.027	1	0.084	0.965^**^
	*p*	0.812	–	0.451	< 0.001
	*N*	83	83	83	83
Relative citations 2017–2022	*r*	0.687^**^	0.084	1	0.084
	*p*	< ,001	0.451	–	0.450
	*N*	83	83	83	83
Relative weighted *h*-index 2017–2022	*r*	−0.027	0.965^**^	0.084	1
	*p*	0.812	< 0.001	0.450	–
	*N*	83	83	83	83

^**^Statistically significant coefficient at the level of *p* < 0.01.

**Table 4:** Correlations between metrics of ESS scientists of the AD of M-EDF/01 and M-EDF/02.

		**Total citations 2017–2022**	**Total weighted *h*-index 2017–2022**	**Relative citations 2017–2022**	**Relative weighted *h*-index 2017–2022**
**AD: physical training and methodology (code M-EDF/01)**					
Total citations 2017–2022	*r*	1	0.645^**^	0.691^**^	0.460^**^
	*p*	.	< 0.001	< 0.001	0.002
	*N*	42	42	42	42
Total weighted *h*-index 2017–2022	*r*	0.645^**^	1	0.525^**^	0.551^**^
	*p*	< 0.001	–	< 0.001	< 0.001
	*N*	42	42	42	42
Relative citations 2017–2022	*r*	0.691^**^	0.525^**^	1	0.530^**^
	*p*	< 0.001	< 0.001	.	< 0.001
	*N*	42	42	42	42
Relative weighted *h*-index 2017–2022	*r*	0.460^**^	0.551^**^	0.530^**^	1
	*p*	0.002	< 0.001	< 0.001	–
	*N*	42	42	42	42
**AD: sport sciences and methodology (code M-EDF/02)**					
Total citations 2017–2022	*r*	1	0.574^**^	0.615^**^	0.339^*^
	*p*	.	< 0.001	< 0.001	0.032
	*N*	41	41	41	41
Total weighted *h*-index 2017–2022	*r*	0.574^**^	1	0.460^**^	0.595^**^
	*p*	< 0.001	–	0.003	< 0.001
	*N*	41	41	41	41
Relative citations 2017–2022	*r*	0.615^**^	0.460^**^	1	0.677^**^
	*p*	< 0.001	0.003	–	< 0.001
	*N*	41	41	41	41
Relative weighted *h*-index 2017–2022	*r*	0.339^*^	0.595^**^	0.677^**^	1
	*p*	0.032	< 0.001	< 0.001	–
	*N*	41	41	41	41

^**^Statistically significant coefficient at the level of *p* < 0.01.

^*^Statistically significant coefficient at the level of *p* < 0.05.

Analysis of the impact of the scientific output of ESS scientists aggregated by AD showed significant trends in metrics, as shown in Table 3. Significant positive correlations emerged between total citations and relative citations during 2017–22 *(r* = 0.687). Similarly, a significant association was found between total *h*-index and relative *h*-index weighted by authorship (*r* = 0.965). Significant trends emerge in the metrics of the same researchers when analyzed separately by AD, as shown in Table 4.

In order to analyse the correlation between the dependent variable “year” and the independent variables such as “Total citations 2017–22,” “Relative citations 201–22,” “Total weighted *h*-index 2017–22,” and “Relative weighted *h*-index 2017–22,” a non-parametric multivariate linear regression model was used, as shown in Table 5.

**Table 5:** Non-parametric regression analysis.

**Model**	**Unstandardized coefficients**		**Standardized coefficients**	***t***	***p***
	***B***	**Std. Error**	**Beta**		
(Constant)	2.879	0.143		20.157	< 0.001^*^
Total citations 2017–22	0.004	0.001	0.398	6.709	< 0.001^*^
Relative citations 2017–22	0.001	0.001	0.078	1.304	0.193
Total weighted *h*-index 2017–22	0.390	0.124	0.448	3.158	0.002^*^
Relative weighted *h*-index 2017–22	−0.184	0.083	−0.315	−2.224	0.027^*^

Dependent variable: year.

^*^Statistically significant coefficient at the level of *p* < 0.01.

## 4 Discussion

This study aimed to assess the authorship-weighted impact of Italian scientists in the ESS field, within the subfield “Sport Sciences” from 2017 to 2022, through the “Researcher Discovery” function of Scopus. Data analysis reveals that within the group of 200 scientists returned by the Scopus database, only 83, out of a total population of 259, are framed on the ESS. Hence, less than half of the scientists are framed on the ESS. It is important to note that among the 200 scientists identified in the Scopus database, there are 117 experts from other AD, or not framed in any AD because they are outside the university faculty, who produce scientifically relative to the “Sport Sciences” subfield.

The analysis of the impact of the scientific production of ESS scientists aggregated by AD showed significant trends in metrics, as shown in Table 3. These results are particularly significant as they not only highlight the contributions of Italian scientists to the field but also provide a framework for understanding the dynamics of research impact within a specialized area. Establishing a positive association between total production and impact with the relative one underscores the relevance of localized research efforts in the broader academic landscape. This is clear evidence that the 83 Italian ESS scientists among the 200 proportionally produce and impact between overall research and that related to the subfield of Sport Sciences. This finding emphasizes the innovative nature of the research output from this group, suggesting that even those outside traditional academic settings can significantly influence the field. The study's ability to aggregate data across diverse academic disciplines presents a novel approach to evaluating scientific output and impact. Moreover, although less than half of the Italian scientists are framed within the ESS, they produce a correlated trend between total impact and that related to the subfield “Sport Sciences”. The study's ability to aggregate data across academic disciplines (AD) presents a novel approach to evaluating scientific output and impact. The identification of significant trends in metrics is essential, as it illustrates the interconnectedness of various research outputs and their implications for the subfield. Significant trends emerge in the metrics of the same scientists when analyzed separately for AD, as shown in Table 4. Both AD show significant correlation coefficients (*p*-value < 0.01) between the variables considered, but there are small differences in the specific values of the correlation coefficients. The implementation of a regression analysis allowed for a more detailed exploration of the influence of the independent variables—Total citations 2017–22, Relative citations 2017–22, Total weighted *h*-index 2017–22, Relative weighted *h*-index 2017–22—on the dependent variable “year” The decision to use this variable as the dependent variable was driven by the intent to analyze in detail how the independent variables can influence the observed changes during the period from 2017 to 2022. This approach allows for monitoring the trends of metric indicators over time, providing a clear view of emerging patterns. However, some criticisms are acknowledged regarding this choice. Using “year” as the dependent variable might not capture all the complexities of variations in the data, as external factors or specific events could influence the results in ways not directly related to the year.

The regression results suggest that each of the specified independent variables has an impact on the dependent variable “year”. In summary, variations in total citations and in the total and weighted relative *h*-index can be interpreted as indicators that can predict or explain the observed changes in the period 2017–2022. This predictive capability adds a significant dimension to the study, offering a methodological innovation that can inform future research assessments and funding decisions within the field. These variables seem to have a predictive or explanatory role regarding the variations recorded over time. Although the other independent variables show a significant association with the year, relative citations do not appear to contribute significantly to the explanation of the temporal variations in the dependent variable.

Among the potential limitations of the study, it is essential to consider several aspects. Firstly, it is important to emphasize that the limit of 200 authors returned by the database for each keyword could compromise the completeness and representativeness of the data obtained. This limitation highlights the need for comprehensive data collection methods in future studies, ensuring a more inclusive representation of the scientific community. This restriction could indeed limit the ability to capture the entire range of authors and publications relevant to our field of research. Therefore, there is a need to conduct further studies aimed at analyzing the scientific output of all 259 ESS scientists. Additionally, it is necessary to consider the potential specific issues associated with the Scopus search system (Gusenbauer and Haddaway, 2020). These could include errors in metadata, the presence of duplicates, or the lack of updates on certain publications, which could affect the accuracy and reliability of the results obtained through the search. Addressing these limitations will be crucial for enhancing the robustness of future research in this area and for ensuring that the contributions of all relevant scientists are adequately represented.

## 5 Conclusions

The detailed analysis of the subfield Sport sciences allowed for an accurate assessment of the impact of the scientific production of the ESS members, weighted by authorship. The analysis revealed the presence of positive and significant relationships between citations and the total *h*-index weighted for the same indicators related to the subfield Sport sciences, both for corresponding and cross-referenced indicators. This result is of particular importance in the study, as it indicates that the overall production and impact follow the trend of production and impact related to the subfield. This suggests that the 83 Italian ESS scientist among the 200 proportionally produce and impact both overall research and the research related to the keywords constituting the subfield “Sport Sciences”. This study is further evidence that the field of ESS is developing consistently, and it suggests that more in-depth studies could measure progress in impact with greater detail.

## Data Availability

The raw data supporting the conclusions of this article will be made available by the authors, without undue reservation.
